# Supplementary material for: Novel QTL Associated with Shoot Branching Identified in Doubled Haploid Rice (Oryza sativa L.) under Low Nitrogen Cultivation
Source: Genes (Basel). 2021 May 14;12(5):745. doi: 10.3390/genes12050745 (PMC8157147; doi:10.3390/genes12050745)
Supplement: Supplementary file 1 [file genes-12-00745-s001.zip › genes-1199924-supplementary.pdf]

genes

MDPI

Novel QTL Associated with Shoot Branching Identified in Doubled Haploid Rice (*Oryza sativa* L.) under Low Nitrogen Cultivation

Youngho Kwon †, Nkulu Rolly Kabange †, Ji-Yun Lee, So-Myeong Lee, Jin-Kyung Cha, Dongjin Shin, Jun-Hyeon Cho, Ju-Won Kang, Jong-Min Ko, and Jong-Hee Lee *

[1] Department of Southern Area Crop Science, National Institute of Crop Science, RDA, Miryang 50424, Korea; kwon6344@korea.kr (Y.H.); olivetti90@korea.kr (S.-M.L.); minitia@korea.kr (J.-Y.L); jknzz5@korea.kr (J.-K.C.); jacob1223@korea.kr (D.S.); hy4779@korea.kr (J.-H.C.); kangjw81@korea.kr (J.-W.K.); kojmin@korea.kr (J.-M.K.);

* Correspondence: Jong-Hee Lee, ccriljh@korea.kr, Tel.: +82-53-350-1168, Fax: +82-55-352-3059

† These authors contributed equally to this work as first authors

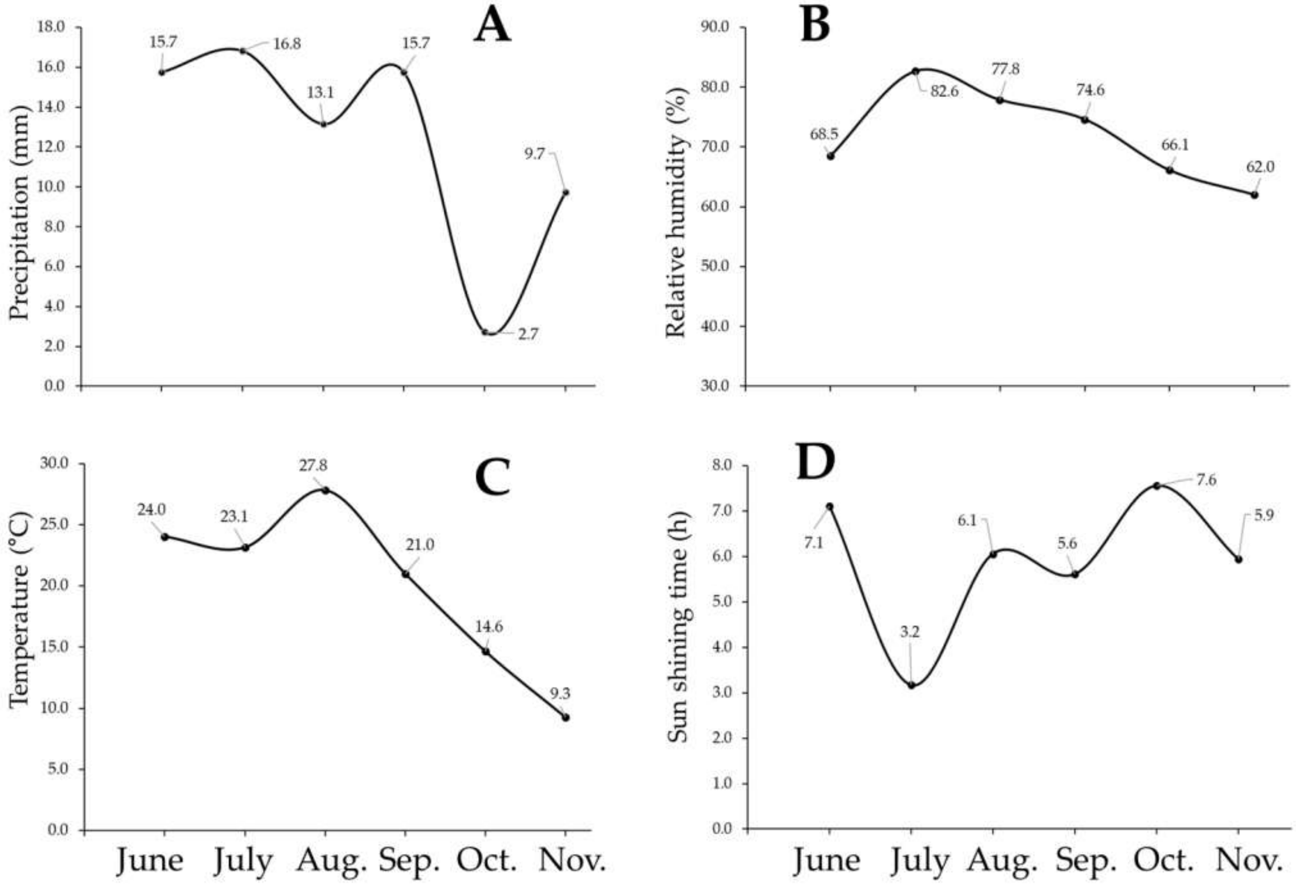

Figure S1. Changes in weather parameters during the rice cropping season of 2020. (**A**) Monthly average precipitation (in mm), (**B**) Monthly average relative humidity (RH), (**C**) Changes in temperature, and (**D**) Pattern of the duration of sun shining (in hours). Data are monthly mean value, obtained from the virtual database of the Korean Meteorological Agency (KMA) (https://data.kma.go.kr).

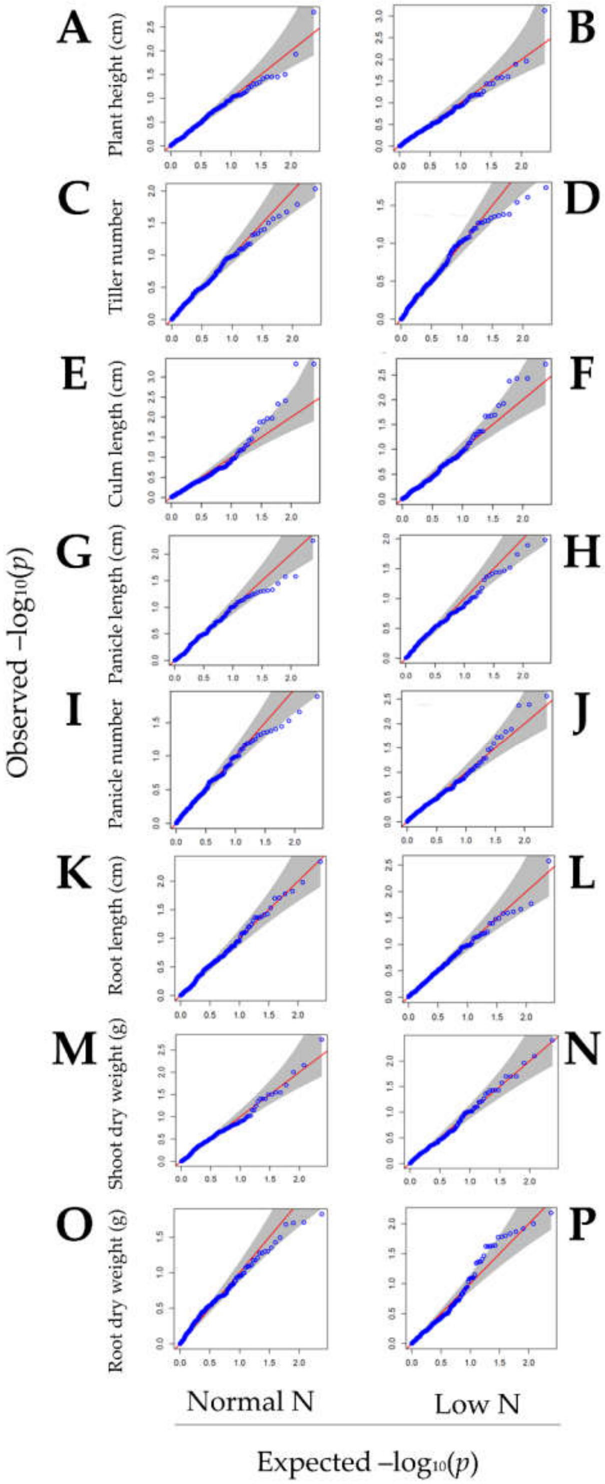

Figure S2. Quantile–Quantile plots. Quantile–Quantile (Q–Q) plots for plant height (**A**,**B**) and number of tillers under normal (**C**,**D**), culm length (**E**,**F**), panicle length (**G**,**H**), panicle number (**I**,**J**), root length (**K**,**L**), shoot (**M**,**N**) and root (**O**,**P**) dry weights under normal and low N cultivation conditions. $-\log_{10}(p)$ is the logarithm base 10 quantile–quantile (Q–Q) of the *p*-values (expected and observed) for traits.

Table S1. Person r correlation between traits under normal nitrogen cultivation

	PHN	TNN	CLN	PLN	PNN	RLN	SDWN	RDWN
PHN		-0.465***	0.150 ns	0.149 ns	-0.437***	0.000 ns	0.350***	-0.027 ns
TNN	-0.465***		0.085 ns	-0.199*	0.603***	0.046 ns	0.317***	0.173 ns
CLN	0.150 ns	0.085 ns		0.299***	-0.052 ns	0.033 ns	-0.005 ns	0.081 ns
PLN	0.149 ns	-0.199*	0.299***		-0.343***	-0.033 ns	-0.088 ns	0.059 ns
PNN	-0.437***	0.603***	-0.052 ns	-0.343***		-0.161 ns	0.109 ns	-0.083 ns
RLN	0.000 ns	0.046 ns	0.033 ns	-0.033 ns	-0.161 ns		-0.117 ns	0.209*
SDWN	0.350***	0.317***	-0.005 ns	-0.088 ns	0.109 ns	-0.117 ns		0.309***
RDWN	-0.027 ns	0.173 ns	0.081 ns	0.059 ns	-0.083 ns	0.209 *	0.309***	

Table S2. Person r correlation between traits under low nitrogen cultivation

	PHL	TNL	CLL	PLL	PNL	RLL	SDWL	RDWL
PHL		-0.279**	0.179 ns	0.071 ns	-0.413***	0.074 ns	0.374***	0.009 ns
TNL	-0.279**		0.198*	-0.222*	0.560***	0.003 ns	0.583***	0.288**
CLL	0.179 ns	0.198 ns		-0.005 ns	-0.058 ns	0.068 ns	0.263**	0.167 ns
PLL	0.071 ns	-0.222 ns	-0.005 ns		-0.343***	-0.009 ns	-0.140 ns	0.043 ns
PNL	-0.413***	0.560***	-0.058 ns	-0.343***		-0.066 ns	0.126 ns	-0.091 ns
RLL	0.074 ns	0.003 ns	0.068 ns	-0.009 ns	-0.066 ns		0.238**	0.440***
SDWL	0.374***	0.583***	0.263**	-0.140 ns	0.126 ns	0.238**		0.491***
RDWL	0.009 ns	0.288**	0.167 ns	0.043 ns	-0.091 ns	0.440***	0.491***	

Plant height under normal nitrogen cultivation (PHN), tiller number under low nitrogen availability (TNL), tiller number under normal nitrogen availability (TNN), culm length under low nitrogen level (CLL), culm length under normal nitrogen level (CNN), panicle length under normal nitrogen cultivation (PLN), panicle number under low nitrogen level (PNL), panicle number under normal nitrogen cultivation (PNN), root length under low nitrogen level (RLL), shoot dry weight under normal nitrogen cultivation (SDWN), and root dry weight under normal nitrogen cultivation (RDWN). *$*p<0.05$*, *$**p<0.01$*, *$***p<0.001$*, *ns* non-significant.

Table S3. Shapiro Wilk W-statistic for the test of normality of the distribution

No.	Trait name	Sample Size	Mean	Variance	S.E	Skewness	Kurtosis	Min.	Max.	Range	*P*-value
1	PHL	117	53.15	32.11	5.67	−0.09	−0.17	40	67.9	27.9	0.568
2	PHN	117	52.5	33.87	5.82	−0.06	−0.47	38.7	65.4	26.7	0.234
3	TNL	117	15.47	15.85	3.98	0.72	0.08	7.7	28	20.3	0.001
4	TNN	117	15.38	13.41	3.66	0.96	1.10	8.3	28.3	20	0.000
5	CLL	117	65.06	46.17	6.79	−0.19	0.02	48	80	32	0.166
6	CLN	117	64.84	47.41	6.89	0.21	0.26	50.08	86.82	36.74	0.450
7	PLL	117	22.16	4.68	2.16	0.35	0.23	16.4	28.6	12.2	0.456
8	PLN	117	21.97	4.68	2.16	0.48	0.60	17.46	29.7	12.24	0.268
9	PNL	117	10.19	11.07	3.33	1.44	3.18	4	25	21	0.000
10	PNN	117	10.2	2.63	1.62	0.07	0.05	6.2	14.3	8.1	0.500
11	RLL	117	19.99	9.44	3.07	0.01	−0.56	12.9	26.1	13.2	0.078
12	RLN	117	20.74	6.18	2.49	0.04	0.27	14	28.5	14.5	0.904
13	SDWL	117	7.143	2.53	1.59	0.47	0.05	3.7	11.6	7.9	0.094
14	SDWN	117	7.286	1.92	1.38	0.45	−0.55	4.7	10.5	5.8	0.001
15	RDWL	117	2.023	1.28	1.13	1.61	3.46	0.6	6.9	6.3	0.000
16	RDWN	117	2.614	1.05	1.02	0.41	−0.50	0.8	5.2	4.4	0.006

Plant height under normal nitrogen cultivation (PHN), tiller number under low nitrogen availability (TNL), tiller number under normal nitrogen availability (TNN), culm length under low nitrogen level (CLL), culm length under normal nitrogen level (CNN), panicle length under normal nitrogen cultivation (PLN), panicle number under low nitrogen level (PNL), panicle number under normal nitrogen cultivation (PNN), root length under low nitrogen level (RLL), shoot dry weight under normal nitrogen cultivation (SDWN), and root dry weight under normal nitrogen cultivation. S.E : standard error. *P*-value: *p*-value of the W-test (the Shapiro Wilk W-statistic for the test of normality).

Table S4. List of candidate genes located within the *qTNN4-1*

Gene locus	Annotation	Molecular function	Biological process	Cellular component
Os04g54474	Basic leucine zipper (bZIP) family protein; TGAL6	Transcription factor activity, sequence-specific DNA binding	Regulation of transcription, DNA-templated; defense response to bacterium; response to cold	Nucleus
Os04g54680	ULP1 protease family protein, putative, expressed ;paralog to LOC_Os01g02250 RGA-1, putative			
Os04g54790	ELMO/CED-12 family protein, putative, expressed	Nucleotide binding; kinase activity	Protein Modification process	Plastid
Os04g54800	Shikimate kinase, putative, expressed	Kinase activity	Metabolic process	Plastid
Os04g54930	ABC transporter, ATP-binding protein, putative, expressed	Transporter activity;hydrolase activity;protein binding	Response to endogenous stimulus; response to abiotic stimulus;tropism; post-embryonic development;multicellular organismal development	
Os04g55070	Gibberellin 20 oxidase 2, putative, expressed	Catalytic activity		
Os04g55090	Pentatricopeptide repeat (PPR repeat) domain containing protein, putative, expressed; maturation of RBL1			
Os04g55360	Ubiquitin carboxyl-terminal hydrolase domain containing protein, expressed	Hydrolase activity;binding	Protein metabolic process	
Os04g55420	Leucine-rich repeat family protein, putative, expressed		Signal transduction	
Os04g55480	BRCA1-associated protein, putative, expressed; BRCA1 is a human tumor suppressor gene (also known as a caretaker gene) and is responsible for repairing DNA. BRCA1 and BRCA2 are unrelated proteins, but both are	Catalytic activity	Protein modification process	
Os04g55410	FGGY family of carbohydrate kinases, putative, expressed	Kinase activity	Carbohydrate metabolic process;response to biotic stimulus;response to abiotic stimulus	Cytosol
Os04g55420	Leucine-rich repeat family protein, putative, expressed		Signal transduction	
Os04g55480	BRCA1-associated protein, putative, expressed	Catalytic activity	Protein modification process;post-embryonic development	Intracellular
Os04g55510	Zinc finger, C3HC4 type domain containing protein, expressed	Binding		
Os04g55520	AP2 domain containing protein, expressed	Sequence-specific DNA binding transcription factor activity	Biosynthetic process	Nucleus
Os04g55560	AP2 domain containing protein, expressed	Sequence-specific DNA binding transcription factor activity	Cell differentiation;anatomical structure morphogenesis;reproduction;multicellular organismal development	
Os04g55640	Plant-specific domain TIGR01627 family protein, expressed		Carbohydrate metabolic process	Golgi apparatus
Os04g55680	Indole-3-acetate beta-glucosyltransferase, putative, expressed	Transferase activity	Metabolic process	
Os04g55730	Alpha-N-acetylglucosaminidase, putative, expressed	Hydrolase activity	Reproduction;post-embryonic development	
Os04g55750	OsWAK54 - OsWAK short gene, expressed	Kinase activity;nucleotide binding	Anatomical structure morphogenesis;cell growth;carbohydrate metabolic process;cellular homeostasis	
Os04g55760	OsWAK55 - OsWAK receptor-like protein kinase, expressed	Kinase activity	Protein modification process	Plasma membrane
Os04g55940	Sodium/calcium exchanger protein, putative, expressed	Transporter activity	Transport	Membrane;vacuole
Os04g55970	AP2-like ethylene-responsive transcription factor AINTEGUMENTA, putative, expressed	Sequence-specific DNA binding transcription factor activity	Anatomical structure morphogenesis;multicellular organismal development	Nucleus
Os04g56360	Cysteine-rich receptor-like protein kinase 8 precursor, putative, expressed	Kinase activity	Protein modification process	Plasma membrane
